# Broadband Normalized Difference Reflectance Indices and the Normalized Red–Green Index as a Measure of Drought in Wheat and Pea Plants

**DOI:** 10.3390/plants14010071

**Published:** 2024-12-29

**Authors:** Ekaterina Sukhova, Yuriy Zolin, Alyona Popova, Kseniya Grebneva, Lyubov Yudina, Vladimir Sukhov

**Affiliations:** Department of Biophysics, N.I. Lobachevsky State University of Nizhny Novgorod, 603950 Nizhny Novgorod, Russia; n.catherine@inbox.ru (E.S.); uchebnayap.zolin@gmail.com (Y.Z.); silverkumiho@mail.ru (A.P.); grebneva.kseniya01@mail.ru (K.G.); lyubovsurova@mail.ru (L.Y.)

**Keywords:** drought, heat maps, multispectral imaging, RGB imaging, RGB indices, remote sensing, spectral bandwidths

## Abstract

Global climatic changes increase areas that are influenced by drought. Remote sensing based on the spectral characteristics of reflected light is widely used to detect the action of stressors (including drought) in plants. The development of methods of improving remote sensing is an important applied task for plant cultivation. Particularly, this improvement can be based on the calculation of reflectance indices and revealing the optimal spectral bandwidths for this calculation. In the current work, we analyzed the sensitivity of broadband-normalized difference reflectance indices and RGB indices to the action of soil drought on pea and wheat plants. Analysis of the heat maps of significant changes in reflectance indices showed that increasing the spectral bandwidths did not decrease this significance in some cases. Particularly, the index RI(659, 553) based on the red and green bandwidths was strongly sensitive to drought action in plants. The normalized red–green index (NRGI), which was the RGB-analog of RI(659, 553) measured by a color camera, was also sensitive to drought. RI(659, 553) and NRGI were strongly related. The results showed that broadband and RGB indices can be used to detect drought action in plants.

## 1. Introduction

Agriculture is an important human activity that provides food security; however, global climate changes increase areas that are influenced by drought and disrupt plant cultivation. It is known that the action of abiotic adverse factors (particularly, drought) induces the development of stress responses including the disbalance of water and gas exchange, suppression of photosynthetic activity, decrease in content of pigments, and others [1]. These stress responses decrease the growth and productivity of agricultural plants and, thereby, restrict their crop [2,3]. The early detection of stress changes in plants is the basis of timely protective actions (e.g., treatment by hormones and fertilizers [4,5]) which can increase plant sustainability and productivity. The development of this detection is an important task of precise agriculture, which includes plant remote sensing, the analysis of measured data, decision making, and corrective actions [6,7].

Measurements of the spectral characteristics of reflected light can provide extensive information about plants including the parameters of their physiological processes [8,9]. Particularly, plant reflectance in the visible spectral region is dependent on the parameters of photosynthetic processes [10,11] and content of leaf pigments [12,13]. Both photosynthetic processes and the content of pigments play an important role in the growth and productivity of plants and participate in their protection from the action of stressors [2,9,14]; i.e., remote sensing of these characteristics based on leaf or canopy reflectance can be a key step of the plant cultivation. Plant reflectance in the near-infrared spectral region is related to leaf structure; in contrast, reflectance in both the near-infrared and short-wavelength infrared spectral regions is dependent on water content and can be used for the remote sensing of the water exchange in plants [15,16,17].

Multispectral and hyperspectral cameras/detectors are used to measure the spectral characteristics of plant reflectance [9]. These cameras/detectors can be localized on numerous platforms providing different spatial and temporal resolution; handheld devices, tractors, copters, drones, planes, and satellites are used as the platforms for spectral measurements [9,17,18].

As was noted above, the spectral characteristics of plant reflectance can be dependent on photosynthetic processes, concentrations of pigments, leaf structure, and water contents [9]; moreover, changes in these plant characteristics under the action of abiotic stressors, diseases, different regimes of nitrogen nutrition, and other factors can also influence the reflectance of leaves and canopy [18,19,20]. This means that the interpretation of the measured spectral characteristics of plant reflectance can be difficult.

There are approaches that can be used to interpret reflectance measurements, including the analysis of mathematical models to describe light reflectance and transmittance in leaves or canopy (e.g., PROSPECT, [21]) and machine learning to provide a classification of plants in accordance with their characteristics (e.g., revealing stressed and non-stressed plants [22]). The main restriction of these methods is their complexity.

The interpretation of the results of reflectance measurements can also be based on an analysis of reflectance indices (RIs) [8,9] which are a simple and high-throughput tool. Often, RIs are dimensionless indicators, which are calculated on the basis of reflectance in two or three specific bands [9]. Using specific reflectance bands is the basis for improving relations between RIs and specific plant characteristics.

However, remote sensing of plant reflectance in narrow spectral bands requires sophisticated equipment (e.g., hyperspectral cameras localized on drones) [9]; in contrast, the measurement of reflectance in broad bands is based on simple and low-cost cameras, but the efficiency of the broadband RIs can be modified. This problem is also important for the satellite-based monitoring of plant canopy because some satellites can have insufficient spectral resolution [23] and, thereby, cannot measure reflectance indices with narrow spectral bands. As a result, the following question requires further analysis: can RIs based on broad bandwidths be used to estimate plant characteristics?

Literature data show that the efficiency of narrowband reflectance indices is higher than the efficiency of broadband indices [24,25,26] or that both types of RIs are similarly sensitive to plant characteristics [12,25]. Particularly, the bandwidth weakly influences the relationships of indices, which are based on the red and near-infrared (NIR) spectral regions, to the leaf area index and canopy chlorophyll density; in contrast, the positions of the centers of these spectral bands are more important for the efficiency of the indices [25]. On the other hand, Broge and Leblanc showed that broadband RIs, which are used to estimate the leaf area index and canopy chlorophyll density, have lower sensitivity to errors caused by environmental factors in comparison with narrowband indices [12].

Broadband RIs are mainly calculated on the basis of spectral bandwidths equaling tens or hundreds of nm [23,27,28,29]. These indices, which are based on the combination of visible and NIR spectral bands or on only visible bands, can be used to estimate the plant maturing crop [30], nitrogen uptake [31], chlorophyll/carotenoid index [23], chlorophyll content [27,31,32], biomass [27,31], leaf area index [24], evapotranspiration [26], and gross primary production [23]. Additionally, broadband RIs can be used to reveal fast physiological changes in plants that are induced by electrical signals [29,33].

It should be finally noted that there is an additional direction of investigations of the broadband indices: an analysis of the relationships of broadband RIs to color parameters which are shown using red–green–blue (RGB) imaging (the imaging based on the application of color cameras [34]). RGB imaging is the simplest and fastest method of remote sensing of plants [34]; however, the interpretation of its results is very difficult. It is known that color parameters can be used to estimate the growth rate, crop, pigment content, and other plant characteristics [6,8,34]. Revealing the relationships of broadband RIs, which are sensitive to plant characteristics, to RGB indices means that the last indices can also be effectively used for the remote sensing of plants.

In our previous work [35], we complexly analyzed the sensitivity of narrowband RIs to plant changes under water shortage and heating by using heat maps of the significances and directions of changes in these indices. Some sensitive narrowband RIs were shown; however, we did not analyze the influence of spectral bandwidths on the changes in RIs. Particularly, the sensitivity of broadband RIs, which can be measured using simple multispectral cameras [9,34], to the action of the investigated stressors was not analyzed. Thus, the current work was devoted to the analysis of the influence of spectral bandwidths on the sensitivity of normalized difference reflectance indices to drought action in pea and wheat plants including the investigation of the sensitivity of broadband RIs. Additionally, we compared the sensitivity of the broadband RIs and the corresponding RGB indices to this action because RGB imaging is the simplest method of the optical remote sensing of plants [34].

## 2. Results

### 2.1. Influence of Spectral Bandwidth on Sensitivity of Reflectance Indices to the Drought Action in Pea and Wheat Plants

**Figure 1:**
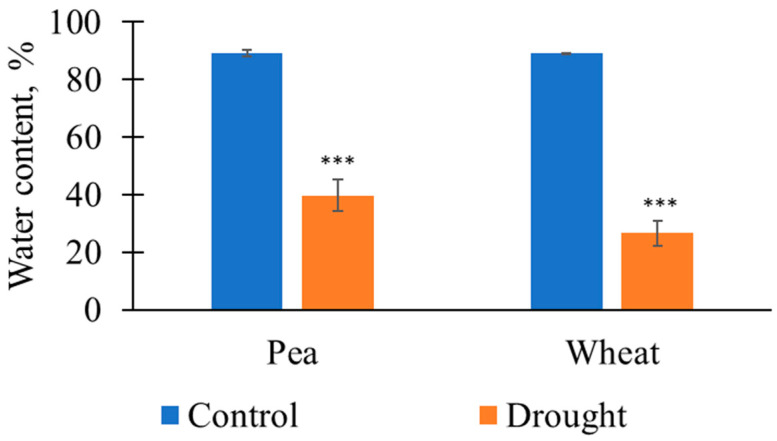
The relative water contents in the pea and wheat plants on the 12th day of soil drought and under control conditions (*n* = 5 for pea plants and *n* = 15 for wheat plants). The control plants were irrigated three times per week (Monday, Wednesday, and Friday); the plants were not irrigated to induce soil drought. The 1st day without irrigation (Monday) was assumed as the 1st day of the drought. ***, the relative water contents significantly differed in the plants under irrigation and under drought (*p* < 0.001).

First, Figure 1 shows that the 12-day soil drought caused a significant decrease in the relative water content in the shoots of the pea and wheat plants in comparison with this content in the control. This decrease showed that the drought duration was enough for large water loss by the plants and could be used in further investigations.

Heat maps showing significant changes in normalized difference reflectance indices under drought (in comparison to control values) and the directions of these changes were used to estimate the sensitivity of RIs to the soil drought action at different spectral bands.

**Figure 2:**
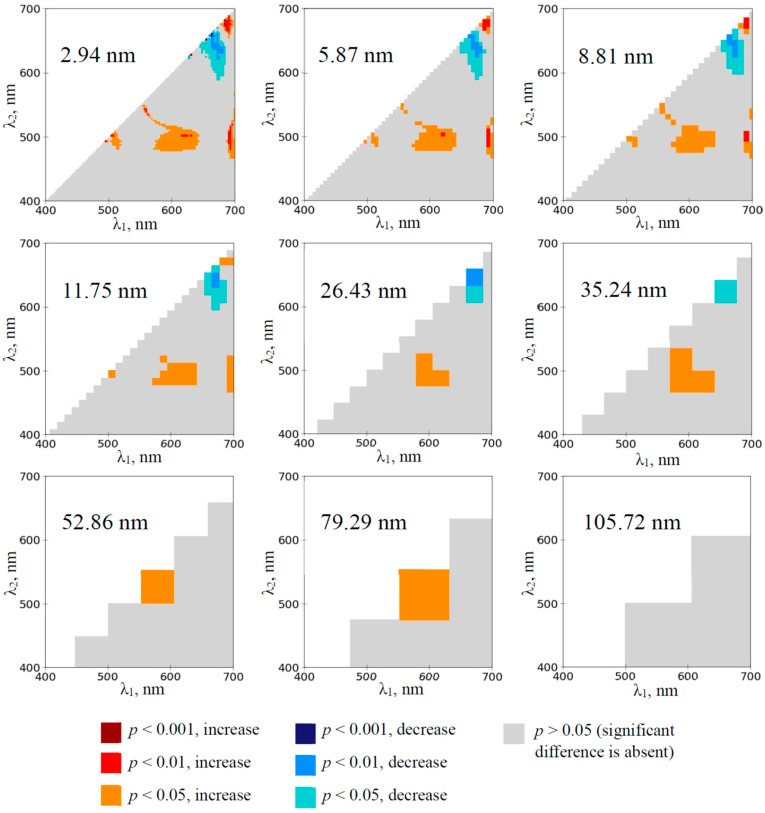
The heat maps of the significance and direction of differences between the normalized difference reflectance indices (RIs) in the pea plants under drought and control conditions on the 1st day of drought (*n* = 10). The spectral bandwidths are shown on maps. The significance and direction of changes in RIs are shown by colors.

Two spectral regions provided significant changes in RIs on the 1st day of drought in pea plants (the 3rd day of absence of irrigation, see Section 4.1 for details) when 2.94 nm bandwidths were used (Figure 2). RIs were (i) significantly increased at λ_1_ and λ_2_ equaling to about 570–640 nm and 480–510 nm, respectively, and (ii) significantly decreased at λ_1_ and λ_2_ equaling to about 650–690 nm and 590–640 nm, respectively. The significantly increased RIs were observed at increasing bandwidth from 2.94 to 79.29 nm. The significantly decreased RIs were observed at increasing bandwidth from 2.94 to 35.24 nm. There were additional small spectral regions providing significant changes in the RIs (Figure 2); however, these regions were absent at increasing bandwidths more than 11.75 nm. Any significant changes in RIs, which were calculated on the basis of 105.72 nm bandwidth, were absent.

**Figure 3:**
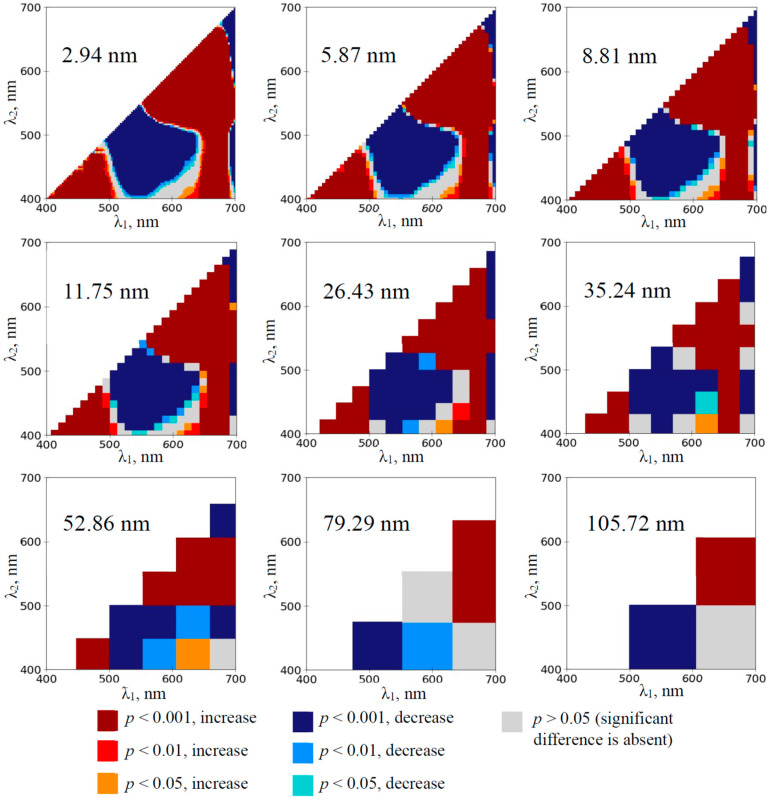
The heat maps of the significance and direction of differences between the normalized difference reflectance indices (RIs) in the pea plants under drought and control conditions on the 12th day of drought (*n* = 10). The spectral bandwidths are shown on maps. The significance and direction of changes in RIs are shown by colors.

The development of the water deficit in the pea plants induced the extension of spectral regions with significant changes in RIs calculated on the basis of 2.94 nm bandwidth (Figures S1–S4). The maximum effect was observed on the 12th day of the soil drought (Figure 3); most normalized difference reflectance indices were significantly changed in this case. Increasing bandwidths decreased the sensitivity of RIs to the drought action in the pea plants; however, spectral regions with increased and decreased RIs were observed at even 105.72 nm bandwidths from the 3rd and 8th days, respectively.

**Figure 4:**
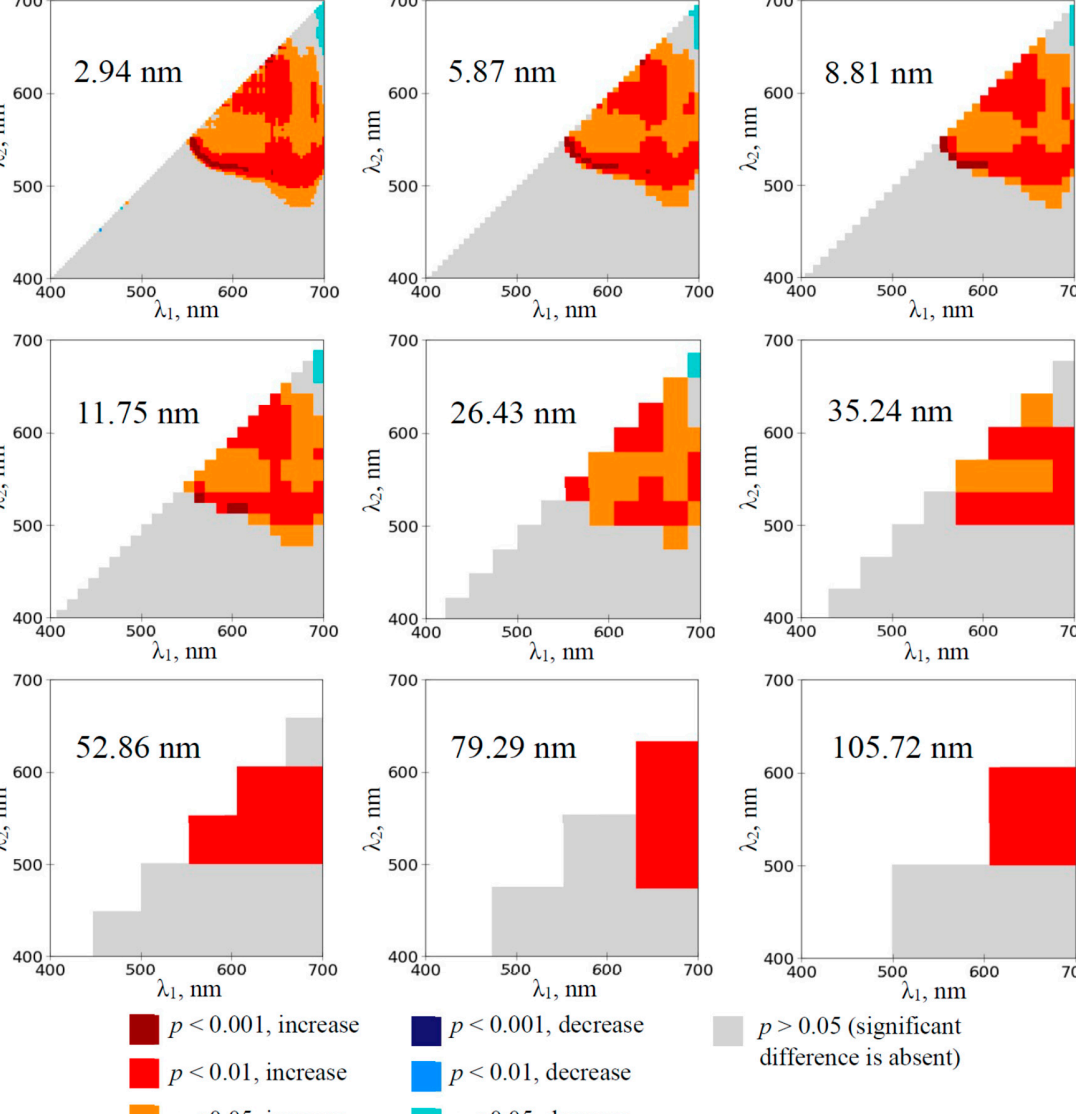
The heat maps of the significance and direction of differences between the normalized difference reflectance indices (RIs) in the wheat plants under drought and control conditions on the 1st day of drought (*n* = 10). The spectral bandwidths are shown on maps. The significance and direction of changes in RIs are shown by colors.

The investigation of the wheat plants showed that RIs, which were calculated on the basis of 2.94 nm bandwidth, were significantly increased at λ_1_ and λ_2_ equaling to about 550–700 nm and 480–650 nm, respectively, on the 1st day of the soil drought (Figure 4). This effect was also observed at increased bandwidths including 105.72 nm bandwidth.

**Figure 5:**
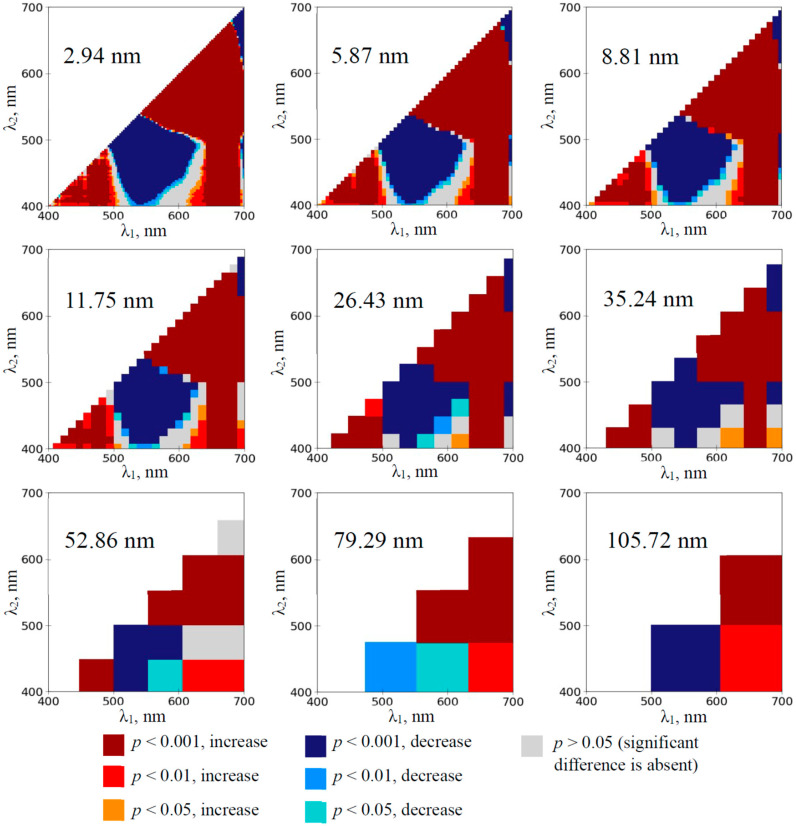
The heat maps of the significance and direction of differences between the normalized difference reflectance indices (RIs) in the wheat plants under drought and control conditions on the 12th day of drought (*n* = 10). The spectral bandwidths are shown on maps. The significance and direction of changes in RIs are shown by colors.

The development of the water deficit in the wheat plants induced the extension of the spectral region with significantly increased RIs (Figures S5–S8); the maximum effect was observed on the 12th day of the soil drought (Figure 5). This effect was observed at all the spectral bandwidths that were used in the investigation (including 105.72 nm bandwidth). The large spectral region with decreased RIs was observed on the 3–12th days of the soil drought at the low bandwidths; in contrast, decreased RI was only observed on the 10th and 12th days at 105.72 nm bandwidth.

**Figure 6:**
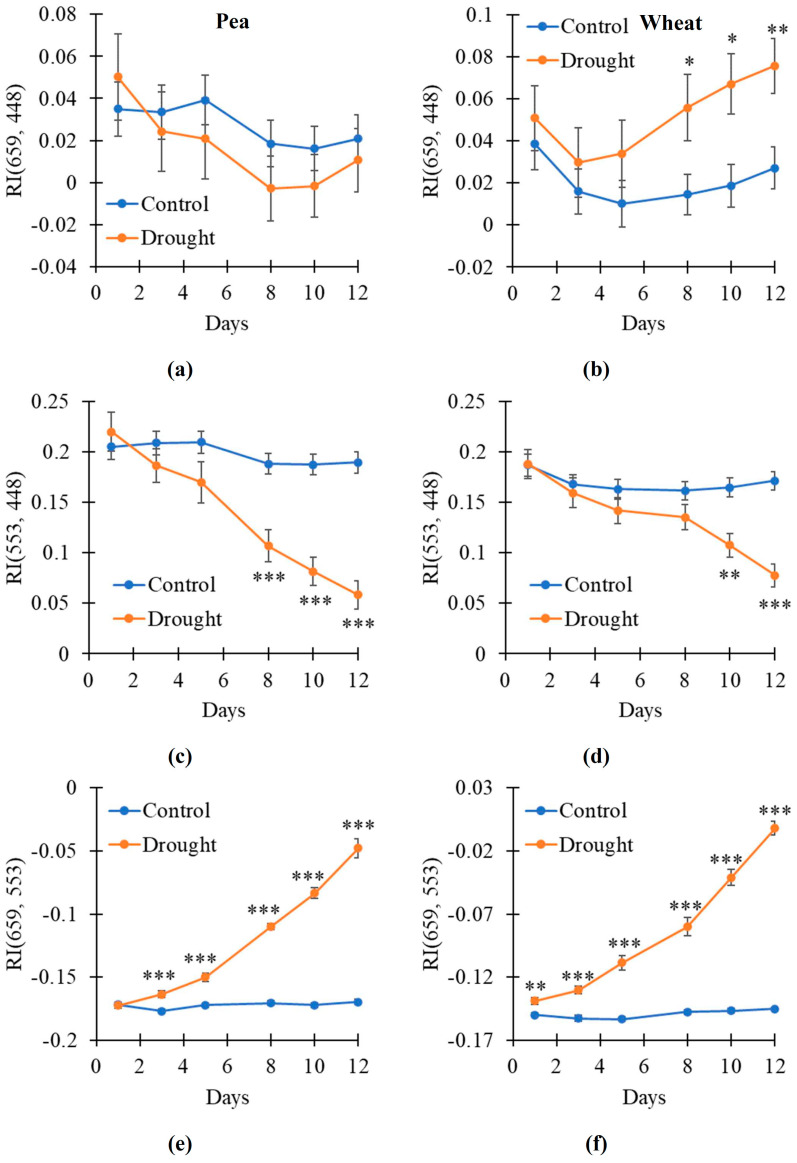
The dynamic of changes in the broadband reflectance indices under drought and control conditions (*n* = 10). Dynamics of RI(659, 448) with 659 nm and 448 nm centers of spectral bands in pea (**a**) and wheat (**b**), RI(553, 448) with 553 nm and 448 nm centers in pea (**c**) and wheat (**d**), and RI(659, 553) with 659 nm and 553 nm centers in pea (**e**) and wheat (**f**) are shown. Only 105.72 nm bandwidths were used. Asterisks show significant differences between the plants under drought and control conditions (*, *p* < 0.05; **, *p* < 0.01; ***, *p* < 0.001).

After that, we investigated the drought-induced changes in the broadband-normalized difference reflectance indices with 105.72 nm bandwidths including RI(659, 448) with 659 nm and 448 nm centers of spectral bands, RI(553, 448) with 553 nm and 448 nm centers, and RI(659, 553) with 659 nm and 553 nm centers. It was shown that RI(659, 448) was not significantly changed in the pea plants under drought conditions (Figure 6a); in contrast, this index was significantly increased in the wheat plants on the 8–12th days of the soil drought (Figure 6b). RI(553, 448) was significantly decreased on the 8–12th days of the soil drought in the pea plants (Figure 6c) and on the 10–12th days in the wheat plants (Figure 6d). RI(659, 553) was significantly increased on 3–12th days of the soil drought in the pea plants (Figure 6e) and on 1–12th days in the wheat plants (Figure 6f).

Scatter plots between broadband RI(659, 448), RI(553, 448), and RI(659, 553) and similar narrowband indices were analyzed to additionally check relationships between broadband and narrowband RIs. It was shown that these indices were strongly related (R^2^ > 0.87, Figure S8) excluding the broadband and narrowband RI(659, 448) in the pea plants.

Thus, our results showed that broadband RI(659, 553) was sensitive to the drought action of the pea and wheat plants because its changes were initiated on the 1st (wheat) or 3rd (pea) days of the soil drought. Other broadband indices (RI(659, 448) and RI(553, 448)) were also changed under the drought action; however, their sensitivity was lower.

### 2.2. Sensitivity of RGB Indices to the Drought Action in Pea and Wheat Plants

In the next stage of the study, we investigated the RGB indices which were normalized difference reflectance indices based on red, green, and blue reflectance bands. Unlike broadband RIs, which were measured using a hyperspectral camera, the RGB indices were measured using a color camera. Normalized red–blue index (NRBI), normalized green–blue index (NGBI), and normalized red–green index (NRGI), which approximately corresponded to broadband RI(659, 448), RI(553, 448), and RI(659, 553), respectively, were investigated.

**Figure 7:**
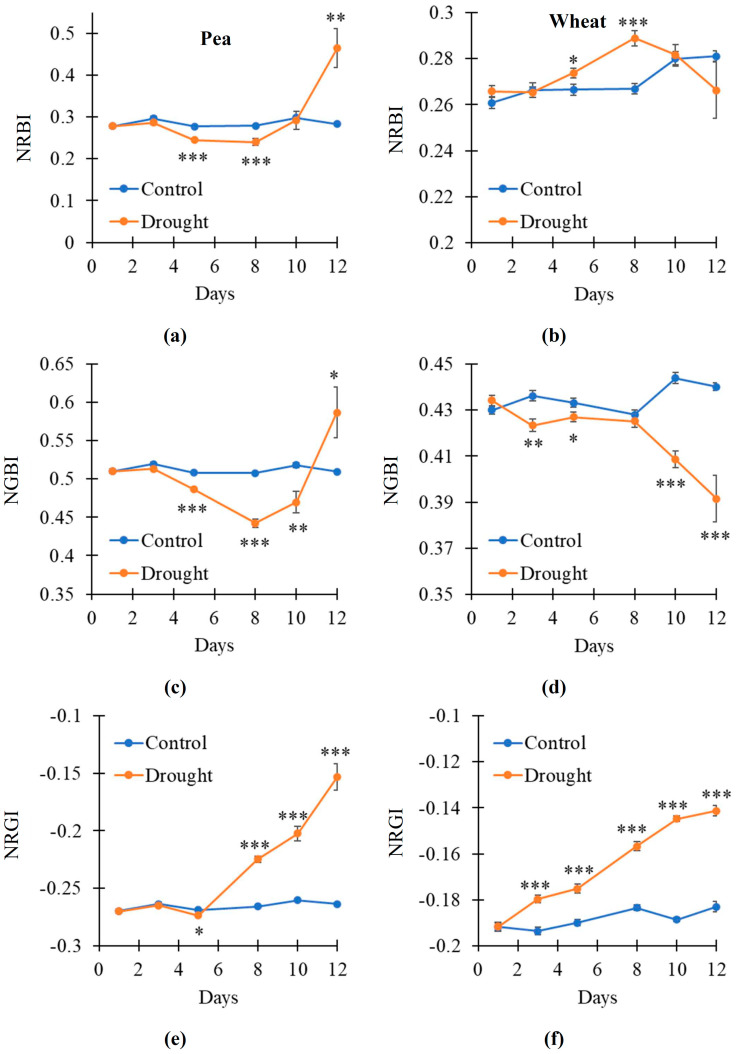
The dynamic of changes in the red–green–blue (RGB) indices under drought and control conditions (*n* = 10). Dynamics of the normalized red–blue index (NRBI) in pea (**a**) and wheat (**b**), normalized green–blue index (NGBI) in pea (**c**) and wheat (**d**), and normalized red–green index (NRGI) in pea (**e**) and wheat (**f**) are shown. Asterisks show significant differences between the plants under drought and control conditions (*, *p* < 0.05; **, *p* < 0.01; ***, *p* < 0.001).

It was shown (Figure 7) that the dynamics of the changes in normalized RGB indices were more intricate than the dynamics of the changes in broadband RIs. Particularly, NRBI was decreased on the 5–8th days of the soil drought and increased on the 12th day in the pea plants (Figure 7a); in contrast, this index was increased on the 5–8th days of the drought action in the wheat plants (Figure 7b). NGBI was decreased on the 5–10th days of the soil drought and increased on the 12th day in the pea plants (Figure 7c); this index was decreased on the 3–5th and 10–12th days of the drought action in the wheat plants (Figure 7d). NRGI was weakly decreased on the 5th day of the soil drought and strongly increased on the 8–12th days in the pea plants (Figure 7e); this index was strongly increased on the 3–12th days of the drought action in the wheat plants (Figure 7f).

Thus, the results showed that the RGB indices had lower sensitivity to the drought action than the corresponding broadband-normalized difference reflectance indices. However, NRGI, which corresponded to RI(659, 553), was the most sensitive to plant changes under the soil drought.

### 2.3. Relationships Between Broadband Reflectance Indices and RGB Indices

In the final stage of the study, we additionally investigated the relationships between the broadband-normalized difference reflectance indices and RGB indices using scatter plots between the average values of these indices.

**Figure 8:**
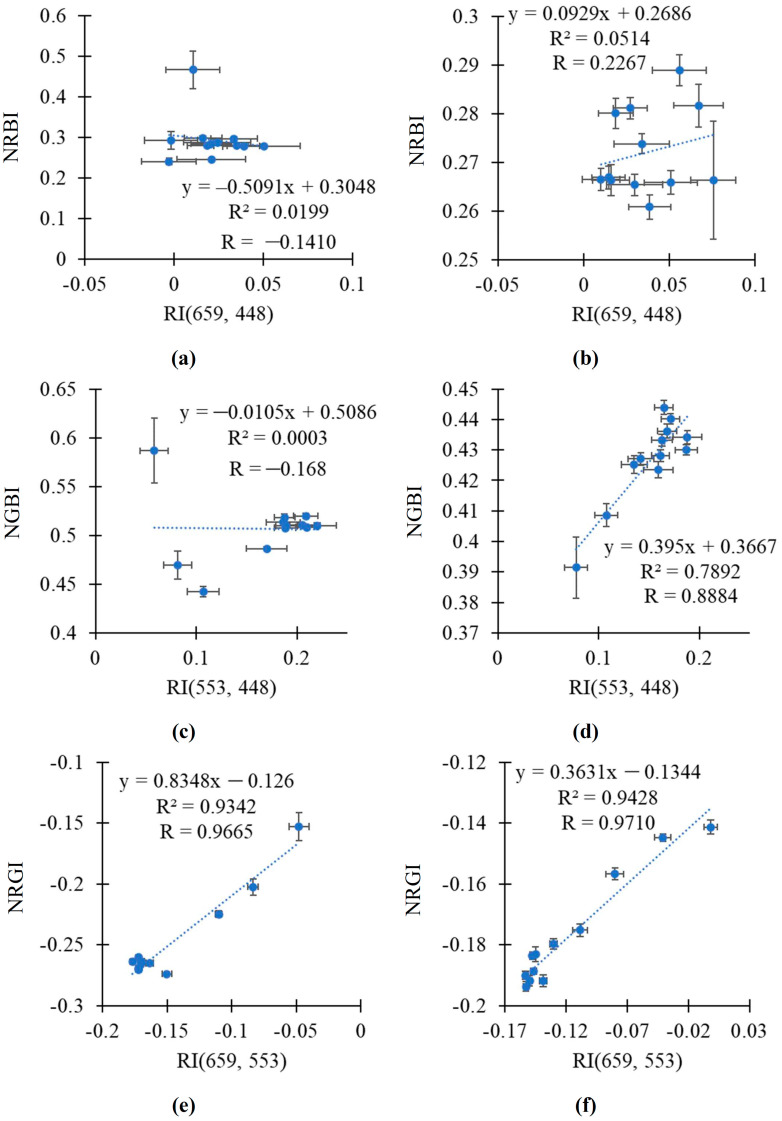
The scatter plots between the broadband-normalized difference reflectance indices and RGB indices in the pea and wheat plants under drought and control conditions. Scatter plots between RI(659, 448) and NRBI in pea (**a**) and wheat (**b**), between RI(553, 448) and NGBI in pea (**c**) and wheat (**d**), and between RI(659, 553) and NRGI in pea (**e**) and wheat (**f**) are shown. The average values of the indices from Figure 6 and Figure 7 were used. R^2^ and R are the determination and correlation coefficients.

It was shown that the relationship between RI(659, 448) and NRBI was weak in both the pea and wheat plants (Figure 8a,b). RI(553, 448) was strongly related to NGBI in the wheat plants (R^2^ = 0.7892); in contrast, this relationship was weak in the pea plants (Figure 8c,d). Finally, RI(659, 553) was strongly related to NRGI in both the pea and wheat plants (R^2^ > 0.93) (Figure 8e,f).

**Figure 9:**
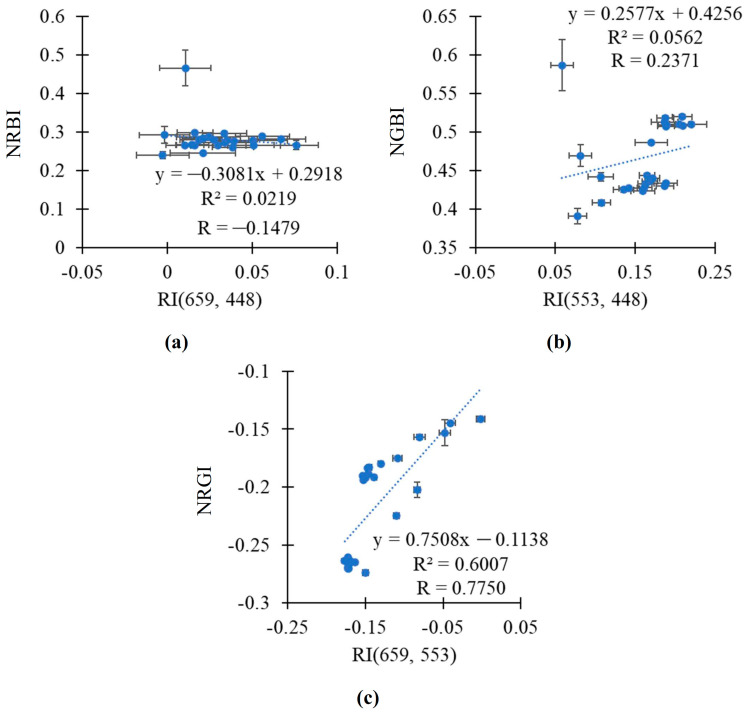
The scatter plots between the broadband-normalized difference reflectance indices and RGB indices based on the total dataset including both the pea and wheat plants under drought and control conditions. Scatter plots between RI(659, 448) and NRBI (**a**), between RI(553, 448) and NGBI (**b**), and between RI(659, 553) and NRGI (**c**) are shown. The average values of the indices from Figure 6 and Figure 7 were used. R^2^ and R are the determination and correlation coefficients.

Relationships between the broadband-normalized difference reflectance indices and RGB indices were additionally investigated on the basis of the total datasets, which included the values of indices in both the pea and wheat plants (Figure 9). Using the total datasets decreased relationships in all the investigated pairs of indices (RI(659, 448) and NRBI, RI(553, 448) and NGBI, and RI(659, 553) and NRGI). However, RI(659, 553) was also related to NRGI in this case (R^2^ = 0.6007).

Thus, RI(659, 553) was strongly related to NRGI in the analysis of values in the pea plants or in the wheat plants; linear regression equations accurately describing the dependences of NRGI on RI(659, 553) were proposed. The efficiency of the linear regression equation for the simultaneous analysis of these indices in the pea and wheat plants was lower.

## 3. Discussion

Remote sensing of plants based on optical methods including the reflectance measurements of leaves and canopy is a powerful tool for precise agriculture [9,17,18]. Particularly, it is known that changes in visible light reflectance are mainly caused by changes in absorption by leaf pigments [16,17] which play a key role in plant energy exchange and metabolism [36], protection of photosynthetic machinery [14], and regulation of physiological processes [37,38]. Reflectance in the near-infrared and short-wavelength infrared spectral regions is related to leaf structure and water content [15,16,17]; i.e., it can also show growth and physiological processes in plants.

Reflectance indices are a very effective tool for the fast interpretation of results of reflectance measurements and estimation of plant characteristics [7,8,39]. However, the effective use of RIs requires revealing optimal bandwidths to calculate these indices because sophisticated systems are necessary for the measurements of narrow reflectance bands [9]; in contrast, the informativity of RIs can be decreased by using broad bandwidths [24,25,26] which are measured by more accessible systems. Thus, our current work is devoted to the investigation of the sensitivity of broadband-normalized reflectance indices and RGB indices to the action of the soil drought on pea and wheat plants.

First, the analysis of heat maps of the significance and direction of changes in RIs under the drought action shows that the development of the soil drought induces changes in a large number of RIs mainly localized in two spectral regions on heat maps (Figure 2, Figure 3, Figure 4 and Figure 5 and Figures S1–S8): (i) with approximately red λ_1_ and green–yellow λ_2_ (RIs are increased) and (ii) with approximately green–yellow λ_1_ and blue–green λ_2_ (RIs are decreased). RI changes in the first spectral region are initiated earlier (in 1–3rd days) than RI changes in the second region (in 3–5th days) (Figure 2, Figure 4 and Figures S1, S5 and S6).

These spectral regions are probably related to light absorption by chlorophylls and carotenoids [9]. Particularly, the maximums of light absorption by chlorophylls are 431, 663, 458, and 646 nm [40,41]. It is known that drought induces the inhibition of photosynthetic activity and the degradation of chlorophylls [2], it should decrease light absorption and increase reflectance in the red spectral region. In contrast, carotenoids absorb light in approximately 400–500 nm spectral regions [21,40]. It is known that carotenoids play an important role in protective mechanisms including nonphotochemical quenching [14] or the inactivation of reactive oxygen species [42]; i.e., their total concentrations can be increased under the action of stressors [43,44,45] and, thereby, provide an increase in light absorption and decrease in reflectance in the blue–green spectral region. It is also known that the degradation of chlorophylls under the action of stressors can be faster than the degradation of carotenoids [46,47,48]; alternatively, these rates can be similar [48,49]. Different rates of the degradation of chlorophylls and carotenoids should additionally provide different ratios of light absorption and reflectance in the red and blue spectral regions. Thus, changes in ratios between the concentrations of photosynthetic pigments caused by adaptive responses and/or different rates of their degradation can be reasons for increasing and decreasing reflectance indices in different spectral regions of heat maps, which are shown in the current work.

It should be noted that there are additional mechanisms of changes in reflectance in the green–yellow spectral regions [9], which can also participate in changes in RIs under drought action. It is known that the action of stressors can decrease the reflectance of the green light, with a maximum at about 526–530 nm [50,51,52,53], which is caused by transitions in the xanthophyll cycle, and with a maximum at about 540–545 nm [52], which can be caused by chloroplast shrinkage and/or changes in the aggregation of the light-harvesting complex of photosystem II [14,39,54]. Reflectance at these wavelengths is used to calculate typical [51,52] and modified [55,56] photochemical reflectance indices, which are sensitive to the action of numerous stressors including drought. As a result, the noted mechanisms of decreasing reflectance in the green spectral region can also form the changes in RIs shown in the current work.

Second, our results show (Figure 2, Figure 3, Figure 4 and Figure 5 and Figures S1–S8) that widening spectral bands weakly influence significantly increasing RIs in the first spectral region (with approximately red λ_1_ and green–yellow λ_2_). Particularly, increased RI(659, 553) in this spectral region is observed since the 1st (wheat) or 3rd (pea) days of the soil drought using 105.72 nm bandwidth (Figure 6e,f). In contrast, widening spectral bands decrease the sensitivity of RIs to the drought action in the second spectral region (with approximately green–yellow λ_1_ and blue–green λ_2_). Particularly, decreased RI in this spectral region is observed since the 8th (pea) or 10th (wheat) days of the soil drought using 105.72 nm bandwidth (Figure 6c,d). We hypothesized that this effect can be related to the spectral areas of both regions. The first spectral region is larger; it means that including opposite changes or non-changed parts into the broad bandwidths is not very probable. In contrast, the second region is smaller (especially, in the early days of the soil drought); it means that including opposite changes or non-changed parts into the broad bandwidths can be probable.

Hypothesis about the key role of the spectral area in the induction of changes in broadband RIs shows some restrictions on using these reflectance indices in plant remote sensing. Considering that the concentrations of photosynthetic pigments are dependent on the action of environmental factors [43,44,45,46,47,48,49,57,58] and that the peaks of light absorption by these pigments [59,60] can be narrower than broad bandwidths (about 100 nm), broadband RIs (as well as RGB indices) should be weakly sensitive to intricate changes in pigment content (e.g., changes in ratio between the contents of chlorophylls a and b). In contrast, specific narrowband RIs are sensitive to these changes [9] through high spectral resolution.

As a result, RI(659, 553) with 105.72 nm bandwidth is most sensitive to the drought action in the pea and wheat plants in our investigation; the efficiencies of RI(553, 448) and, especially, RI(659, 448) are lower. This result is in good accordance with the literature data because some works show the sensitivity of the broadband green–red index to the plant biomass [27], content of chlorophylls and water [27], vegetation fraction [28], and gross primary production [23]. It should be noted that the red bandwidth was 50 or 60 nm, and the green bandwidth was 10, 20, or 80 nm in these investigations; in contrast, our results show the efficiency of using bandwidths equaling to about 100 nm. These broad spectral bands are similar to the RGB spectral bands of color cameras [61,62]. It means that RGB indices [34] can potentially be used as broadband-normalized difference indices.

Third, the analysis of NRBI [63], NGBI [64], and NRGI [32], which approximately correspond to broadband RI(659, 448), RI(553, 448), and RI(659, 553), respectively, shows that these RGB indices have lower sensitivity to the drought action than similar broadband RIs (Figure 7e,f). However, NRGI is relatively sensitive to the drought action because it has stably increased since the 8th (pea) or 3rd (wheat) days of the soil drought. It should also be noted that NRGI is strongly related to RI(659, 553) in both the pea and wheat plants (Figure 8e,f). As a result, NRGI can potentially be used to detect plant changes under the action of the soil drought.

Thus, our results show that widening spectral bands weakly influence the sensitivity of normalized difference reflectance indices, which are calculated on the basis of reflectance in the red and green–yellow spectral regions. Particularly, the sensitivity of the broadband RI(659, 553) with 105.72 nm bandwidth is similar to the sensitivity corresponding to narrowband indices. The sensitivity of NRGI, which is the RGB analog of RI(659, 553), is lower; however, this index is also changed under the soil drought (especially, in wheat plants) and is strongly related to the broadband RI(659, 553). In contrast, sensitivity of other RIs to the drought action is strongly decreased with widening spectral bands; the efficiency of NRBI and NGBI is low.

It should be noted that the practical use of RI(659, 553) and NRGI for plant cultivation requires further laboratory, greenhouse, and/or field investigations. There are several points which are important for this use. First, the following is not clear: can these RIs be sensitive to the action of other stressors in plants? Considering the potential role of changes in the content of photosynthetic pigments in the sensitivity (see above), it can be expected that other environmental factors should also influence RI(659, 553) and NRGI. Particularly, it is known that changes in the content of pigments can be caused by the action of salts [65,66], increased temperatures [67], light with different intensities and spectral composition [57,58,68], etc. However, this supposition requires future checking.

Second, plants of different species have different leaf parameters including the content of photosynthetic pigments [69] and leaf thickness [70]. Considering the relationship of the spectra of leaf reflectance to the thickness and content of photosynthetic pigments [71], it means that non-stressed plants of different species can have different values of RI(659, 553) and NRGI; the dependences of these indices on the intensity of stressor action can also differ. The results of the current work support this point: initial RI(659, 553) (Figure 6a,f) and NRGI (Figure 7e,f) and the magnitudes of their changes under drought differed for the pea and wheat leaves. These differences can complicate using RI(659, 553) and NRGI in field or ecological remote sensing. Probably, the adaptation of the revealed RIs to various plant species and, maybe, the preliminary revealing of these plants in images will be necessary.

Third, we used a light source, hyperspectral camera, and RGB camera with fixed positions in the current work; a white standard was used to calibrate reflected light. As a result, errors in measurements were minimized. In contrast, measurements are not so stable in field or ecological remote sensing because the positions of the light source (sun) and camera can change and influence the angles of the incident and reflected light. It is known [51,72,73] that changes in these angles can strongly modify the reflectance of plant canopy. Long distances between plant canopy and cameras, which are typical for, e.g., drone-based remote sensing, can also influence measurements because background elimination can be weakly effective in this case, and corrections of RIs can be necessary [9]. Additionally, calibration of the reflected light can also be restricted in field or ecological remote sensing, which increases errors. Some of these problems (e.g., changes in the angles of the incident and reflected light) can also be observed in greenhouses with combined illumination (sunlight and artificial light). It can be expected that the standardization of measuring conditions (e.g., using the same time of day) can minimize the noted influencing factors.

In spite of the noted challenges, broadband RIs and RGB indices (including RI(659, 553) and NRGI) have important advantages because they are measured by technically simple and low-cost cameras [34] which can be localized on numerous platforms (from handhold measuring systems to satellites). It means that further development of the methods of using these indices for remote sensing is a topical scientific task.

## 4. Materials and Methods

### 4.1. Plant Cultivation, Drought, and Measurements of Relative Water Content

**Figure 10:**
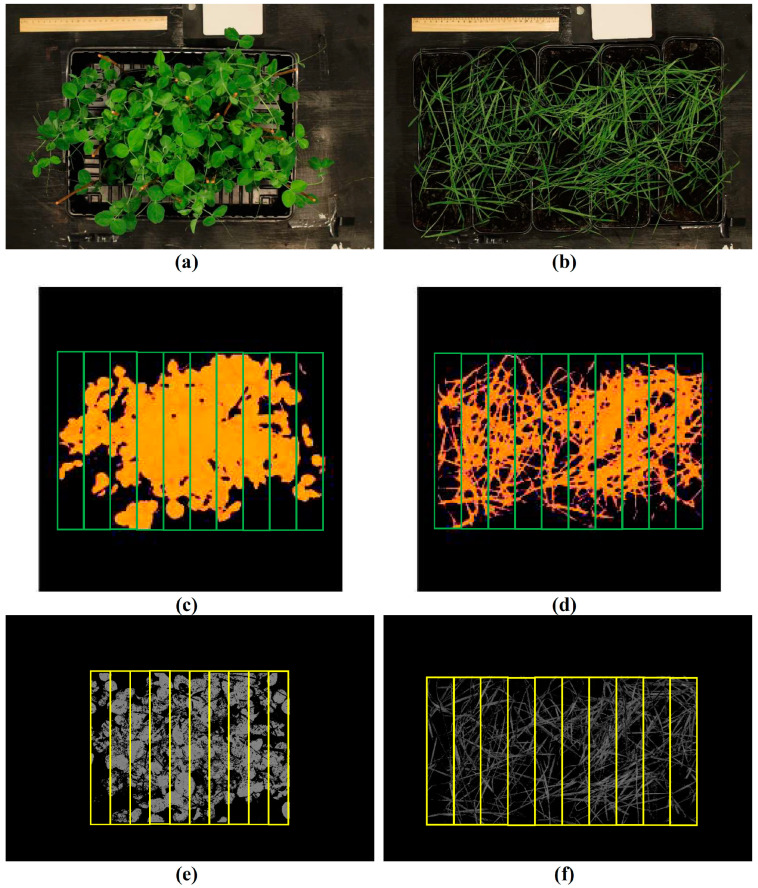
Examples of images for data processing. The RGB images of pea (**a**) and wheat (**b**). The exclusion of background in the hyperspectral images of pea (**c**) and wheat (**d**). The exclusion of background in the RGB images of pea (**e**) and wheat (**f**). The yellow and green frames show the investigated areas in the image (regions of interest, ROIs).

Pea (*Pisum sativum* L., cultivar “Albumen”) and spring wheat (*Triticum aestivum* L., cultivar “Daria”) plants were cultivated in a vegetation room for 4 weeks. The plants were illuminated by luminescent lamps FSL YZ18RR (Foshan Electrical and Lighting Co., Ltd., Foshan, China); the photoperiod was 16 h. The temperature was 24 °C. The plants were cultivated in pots with peat soil “Morris Green” (Pelgorskoe M, Ryabovo, Russia); 9 plants were cultivated per pot. The pots were placed on pallets (4 pots per pallet for pea and 15 pots per pallet for wheat) (Figure 10a,b).

The plants were irrigated three times per week (Monday, Wednesday, and Friday). The soil drought was initiated after 2 weeks of plant cultivation. The experimental plants were not irrigated to induce the soil drought; the control plants were irrigated. The 1st day without irrigation (Monday), which corresponded to the 3rd day of the absence of irrigation, was assumed as the 1st day of the drought.

The absence of irrigation decreased the relative water content in the soil, which was measured by the soil moisture meter Fieldscout TRD 250 (Spectrum Technologies Inc., Herndon, VA, USA) with settings for standard soil. The water content (% from the control value) was about 66% on the 1st day, 27% on the 3rd day, and 18% on the 5th day and was about 80% on the 1st day, 31% on the 3rd day, and 24% on the 5th day in the experiments with pea and wheat, respectively. The final water content in the soil (12th day) was about 8 and 17% in the experiments with pea and wheat, respectively. The results showed that the used model of the soil drought provided a large decrease in the water content in the soil.

The total duration of the soil drought was 12 days. There were two reasons to use this drought duration. (i) Our preliminary works [55,74] showed that fast water loss by plants and strong damage to photosynthetic machinery were initiated after about 7 days of the soil drought. (ii) The leaf withering and drying were observed after 12 days of drought restricting further investigation. Thus, it was expected that “severe” water stress had formed since the 8th day of the drought (after the strong water loss in the soil).

The final relative water content in plant shoots was calculated on the basis of fresh (FW) and dry (DW) weights. FW and DW were measured on the 12th day of the soil drought. Shoots of the plants were dried at 100 °C for 4 h using a thermostat TV-20-PZ-K (Kasimov Instrument Plant, Kasimov, Russia). The water content was calculated by Equation (1):

(1) $$Water\;content=\frac{FW-DW}{FW}\cdot 100\%\;$$

### 4.2. Measurements of Reflectance Spectra and Analysis of Hyperspectral Images

The hyperspectral camera Specim IQ (Specim, Spectral Imaging Ltd., Oulu, Finland) and halogen lamps were standardly fixed in the measuring stand above the pallet with the plants; the distance between the plants and the camera was about 1 m. The white standard (Specim, Spectral Imaging Ltd.) was used for each measurement to calibrate the image and to calculate the reflectance.

The “background” pixels were identified on the basis of the values of NDVI (lower than 0.5 for pea and 0.4 for wheat) and excluded (see Figure 10c,d). NDVI was calculated with using Equation (2):

(2) $$NDVI=\frac{R_{NIR}-R_{Red}}{R_{NIR}+R_{Red}},$$

where R_NIR_ and R_Red_ are reflectance at the NIR (780 nm) and red (680 nm) spectral regions.

Each image was divided into 10 ROIs (Figure 10c,d). Reflectance spectra were averaged for each ROI. Different bandwidths including 2.94, 5.87, 8.81, 11.75, 26.43, 35.42, 52.86, 79.29, and 105.72 nm were used in these spectra. The basic 2.94 nm bandwidths were provided by the hyperspectral camera because this spectral step was used to measure the reflectance spectra. Reflectance at other bandwidths was calculated through averaging reflectance in the neighboring spectral bands (from 2 to 36 bands); the total number of analyzed basic spectral bands was 108. A number of averaged spectral bands were selected to provide maximum bandwidths equaling to about 100 nm with centers in about 450, 550, and 650 nm (in accordance with our previous works [29,33]).

These averaged spectra were used to calculate the normalized difference reflectance indices in accordance with Equation (3):

(3) $$RI=\frac{R_{\lambda_{1}}-R_{\lambda_{1}}}{R_{\lambda_{1}}+R_{\lambda_{1}}},$$

where $R_{\lambda_{1}}$ and $R_{\lambda_{2}}$ were reflectance at wavelengths equaling to λ_1_ and λ_2_. λ_1_ and λ_2_ were varied from 400 to 700 nm. RIs were calculated for all λ_1_ > λ_2_

In accordance with the method proposed in our earlier works [35,75], we calculated the significance of the difference between RIs based on each λ_1_ and λ_2_ in the plants under control and drought conditions using a *t*-test. The direction of differences between RIs was also estimated. Heat maps showing the significance and directions of the drought-induced changes in RIs were constructed on the basis of these results.

We separately investigated broadband-normalized difference reflectance indices with 105.72 nm bandwidth, including RI(659, 448) with 659 nm and 448 nm centers of spectral bands, RI(553, 448) with 553 nm and 448 nm centers of spectral bands, and RI(659, 553) with 659 nm and 553 nm centers of spectral bands.

Special programming tools were developed to provide the described analysis. The language Python with libraries spectral, numpy, scipy, and matplotlib was used.

### 4.3. Measurement and Analysis of RGB Images

The plants were photographed on the RGB camera Canon EOS 4000D (Canon Inc., Tokyo, Japan). The focus length was 35, ISO was 100, and mode of white balance was “tungsten” (automatic white balance was turned off). We used the same measuring stand (see Section 4.2) which fixed the camera and halogen lamps above the plants. The distance between the plants and the camera was 1 m.

Equations (4) and (5) were used to exclude background pixels by thresholding method in the pea and wheat plants, respectively. Mask_Pea_, which was more than 115.33 and less than 139.3, and Mask_Wheat_, which was more than 36.39 and less than 140, were used as criteria for “plant” pixels. Other values of Mask_Pea_ and Mask_Wheat_ indicated “background” pixels.

(4) $${Mask}_{Pea}=5\cdot 255\cdot \frac{0.1G}{0.1G+0.1R+B},$$

(5) $${Mask}_{Wheat}=5\cdot 255\cdot \frac{0.1\left(G-R\right)}{0.1G+0.1R+B},$$

Each image was divided into 10 ROIs (Figure 10e,f). Red (R), green (G), and blue (B) reflectance bands were averaged in each ROI and normalized on R, G, and B of the white standard. Further, we calculated the normalized indices NRBI, NGBI, and NRGI using Equations (6)–(8):

(6) $$NRBI=\frac{R-B}{R+B},$$

(7) $$NGBI=\frac{G-B}{G+B},$$

(8) $$NRGI=\frac{R-G}{R+G},$$

NRBI, NGBI, and NRGI were selected for the analysis because these indices approximately corresponded to the broadbands RI(659, 448), RI(553, 448), and RI(659, 553), respectively.

Special programming tools were developed to provide the described analysis. The language Python with libraries spectral, numpy, scipy, and matplotlib was used.

### 4.4. Statistics

Data were evaluated using descriptive statistics. Averages, standard errors, scatter plots, and quantities of repetitions are shown in figures. Student’s *t*-test was used to estimate the significance of differences between the control and experimental plants.

## 5. Conclusions

The current work was devoted to the analysis of the influence of widening spectral bands on the sensitivity of reflectance indices to the drought actions in pea and wheat plants. It was shown that this widening weakly influenced the sensitivity of normalized difference reflectance indices, which were calculated on the basis of reflectance in the red and green–yellow spectral regions. Particularly, the broadband RI(659, 553) with 105.72 nm bandwidths was strongly affected by the drought action. The sensitivity of NRGI, which was its RGB analog, was lower; however, this index was also changed under the soil drought. In contrast, the sensitivity of other reflectance indices to the drought action is strongly decreased with widening spectral bands.

Our results showed that the measurements of reflected light by systems with broad spectral bands or, even, by color cameras could potentially provide the timely detection of plant changes under the action of stressors (particularly, the soil drought) through using broadband RI(659, 553) and NRGI.

Potentially, RI(659, 553) and NRGI can be used to detect stressor-induced plant changes (e.g., by using drone-based measurements), which is the basis of following protective actions in precision agriculture systems. However, this practical use requires the future investigation of the revealed indices to answer the following questions: Can RI(659, 553) and NRGI be sensitive to the action of other stressors? Can these indices be sensitive to the action of stressors in plants of other species? Can RI(659, 553) and NRGI be effective at long distances between plant canopy and cameras and in the absence of simultaneous calibration?

## Data Availability

The original contributions presented in the study are included in the article/Supplementary Materials; further inquiries can be directed to the corresponding author.

## Supplementary Materials

The following supporting information can be downloaded at https://www.mdpi.com/article/10.3390/plants14010071/s1, Figures S1–S4: The heat maps of the significance and direction of difference between the normalized difference reflectance indices (RIs) in the pea plants under drought and control conditions on the 3rd (Figure S1), 5th (Figure S2), 8th (Figure S3), and 10th (Figure S4) days of drought; Figures S5–S8: The heat maps of the significance and direction of difference between the normalized difference reflectance indices (RIs) in the wheat plants under drought and control conditions on the 3rd (Figure S5), 5th (Figure S6), 8th (Figure S7), and 10th (Figure S8) days of drought; Figure S9: The scatter plots between the broadband- and narrowband-normalized difference reflectance indices in the pea and wheat plants under drought and control conditions.
