# Supplementary material for: Broadband Normalized Difference Reflectance Indices and the Normalized Red–Green Index as a Measure of Drought in Wheat and Pea Plants
Source: Plants (Basel). 2024 Dec 29;14(1):71. doi: 10.3390/plants14010071 (PMC11722858; doi:10.3390/plants14010071)
Supplement: Supplementary file 1 [file plants-14-00071-s001.zip › plants-3337811-supplementary.pdf]

Broadband normalized difference reflectance indices and normalized red-green index are sensitive to drought action on wheat and pea

Ekaterina Sukhova, Yuriy Zolin, Alyona Popova, Kseniya Grebneva, Lyubov Yudina, and Vladimir Sukhov

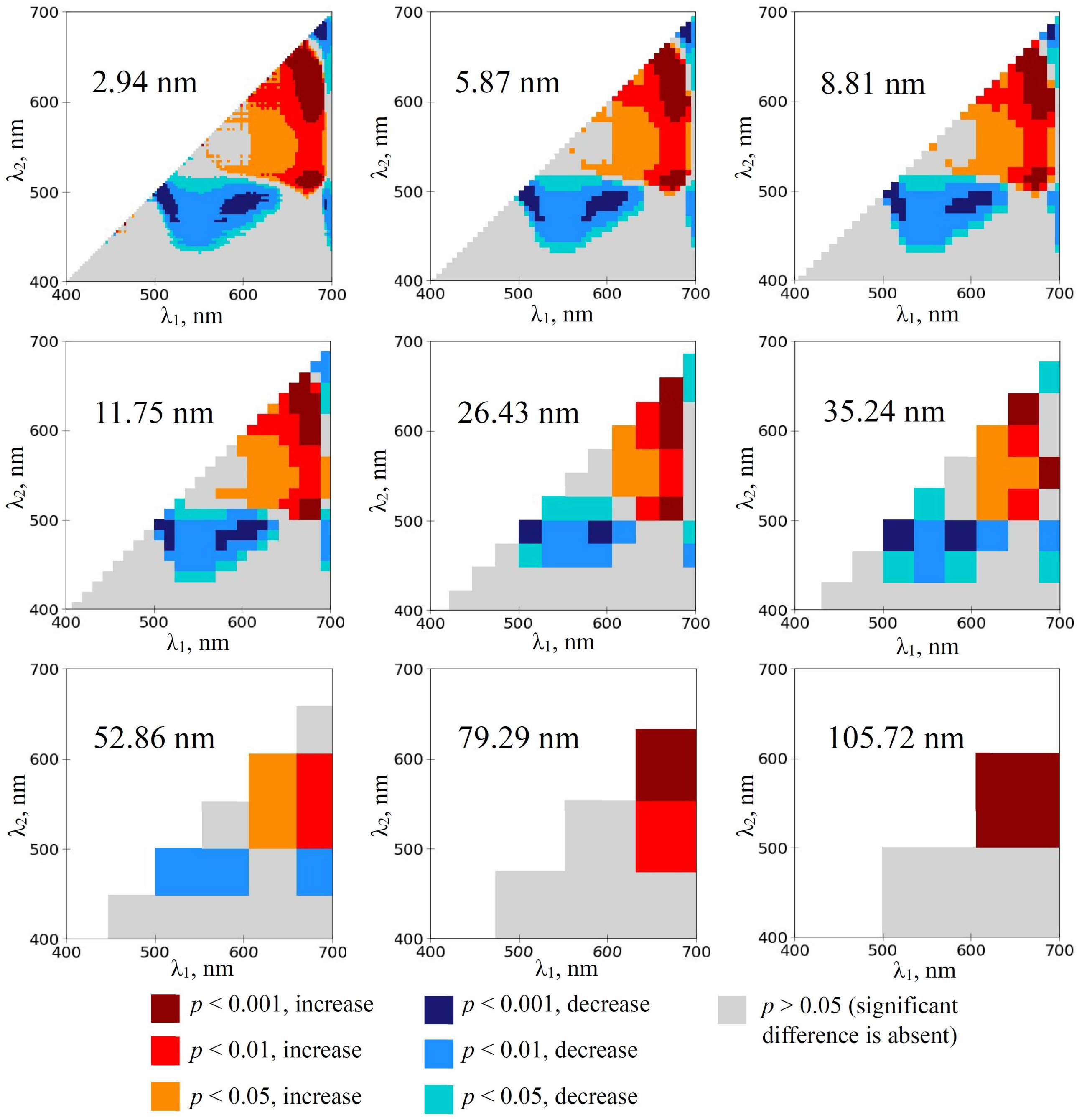

Figure S1. The heat maps of significance and direction of difference between normalized difference reflectance indices (RIs) in pea plants under drought and control conditions on the 3rd day of drought (n=10). The spectral bandwidths are shown on maps. The significance and direction of changes in RIs are shown by colors.

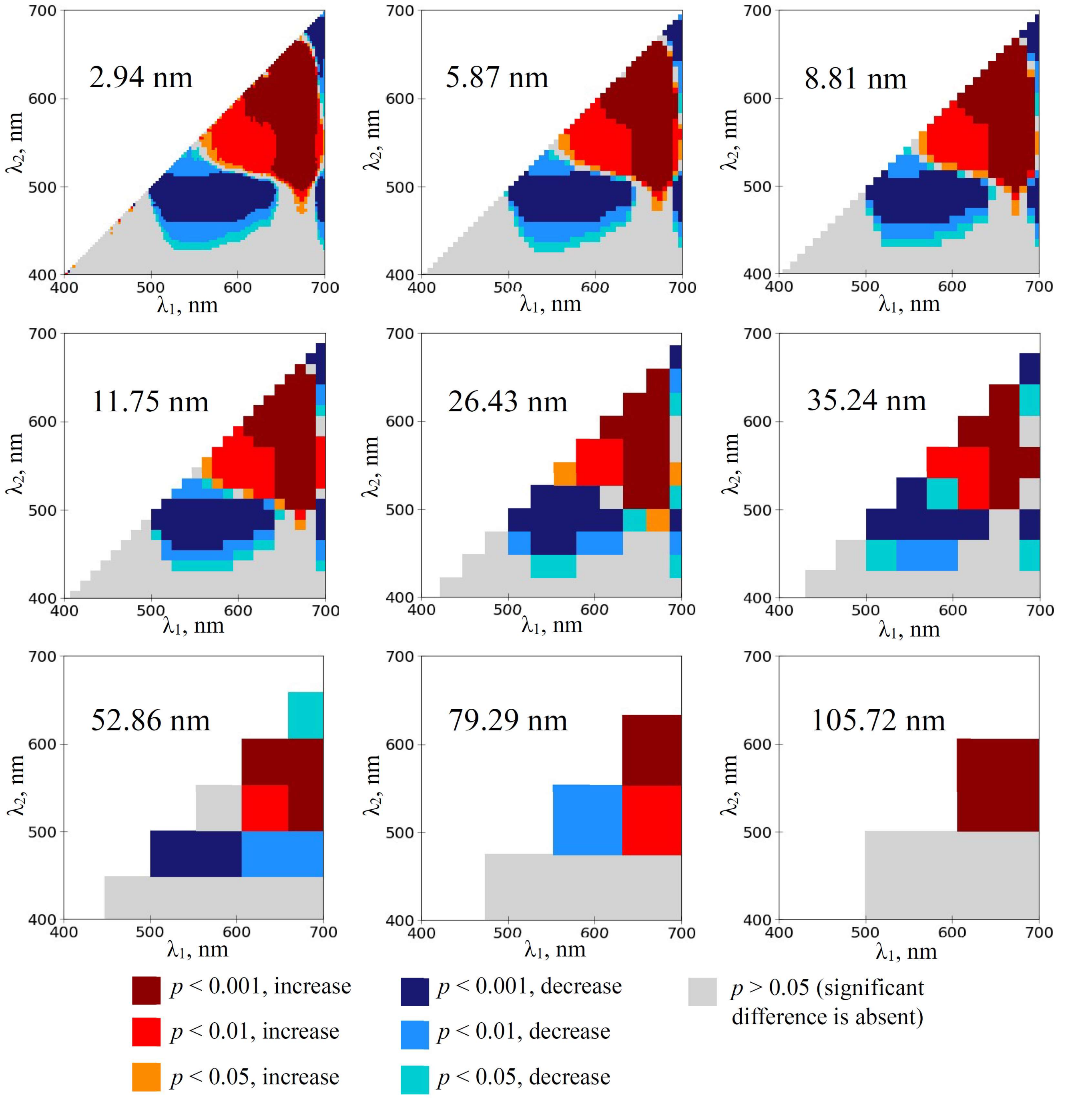

Figure S2. The heat maps of significance and direction of difference between normalized difference reflectance indices (RIs) in pea plants under drought and control conditions on the 5th day of drought (n=10). The spectral bandwidths are shown on maps. The significance and direction of changes in RIs are shown by colors.

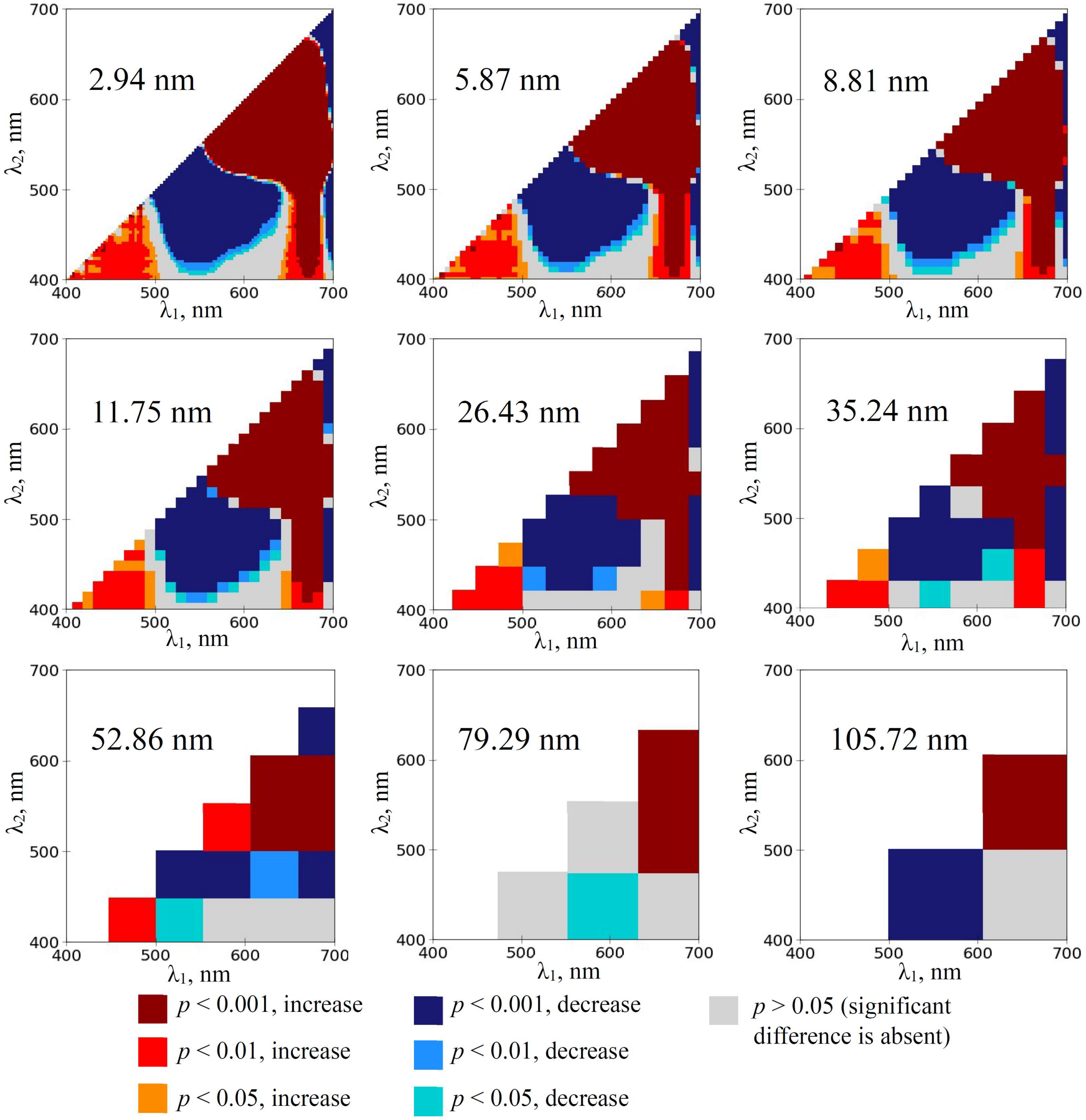

Figure S3. The heat maps of significance and direction of difference between normalized difference reflectance indices (RIs) in pea plants under drought and control conditions on the 8th day of drought (n=10). The spectral bandwidths are shown on maps. The significance and direction of changes in RIs are shown by colors.

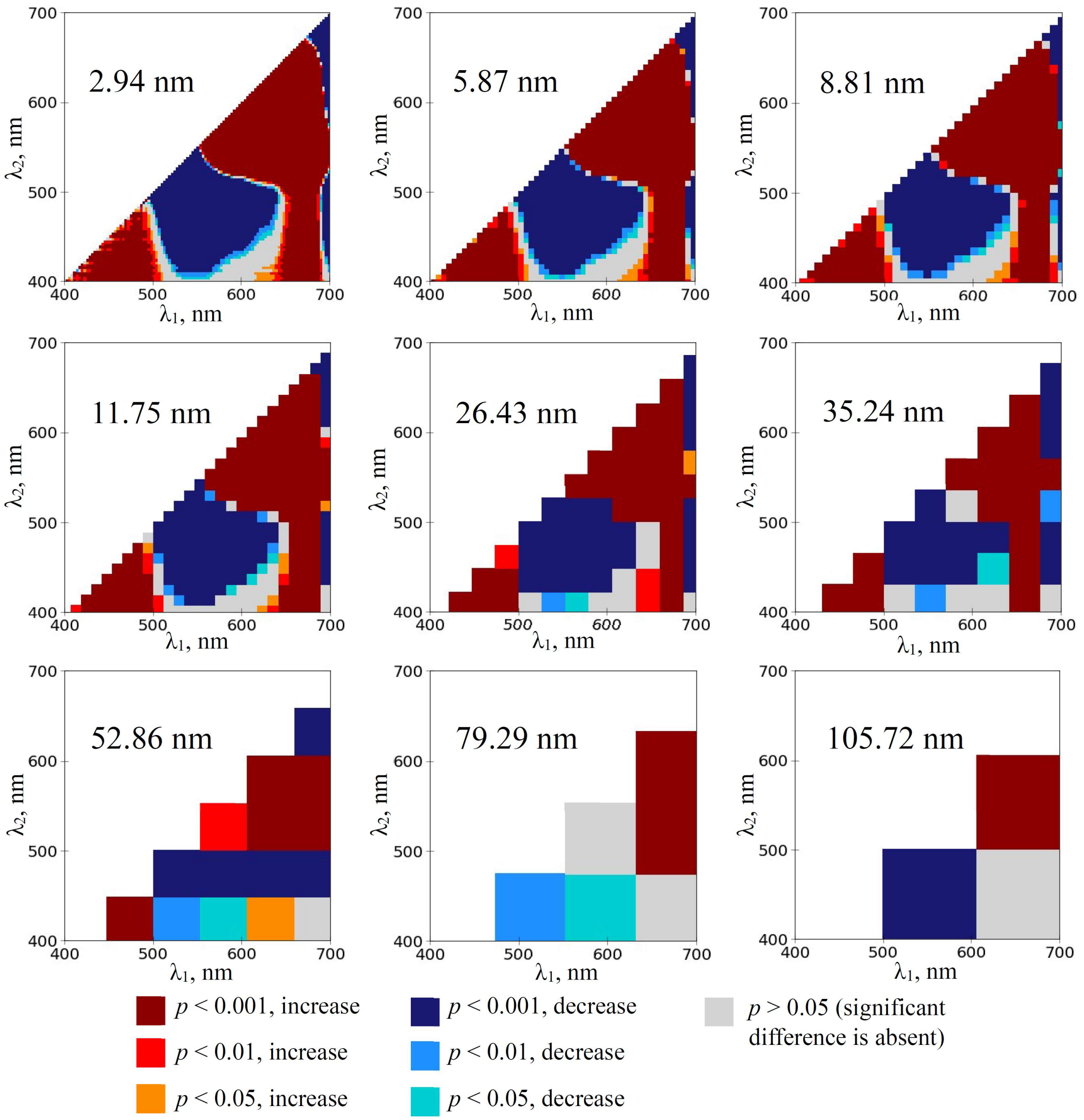

Figure S4. The heat maps of significance and direction of difference between normalized difference reflectance indices (RIs) in pea plants under drought and control conditions on the 10th day of drought (n=10). The spectral bandwidths are shown on maps. The significance and direction of changes in RIs are shown by colors.

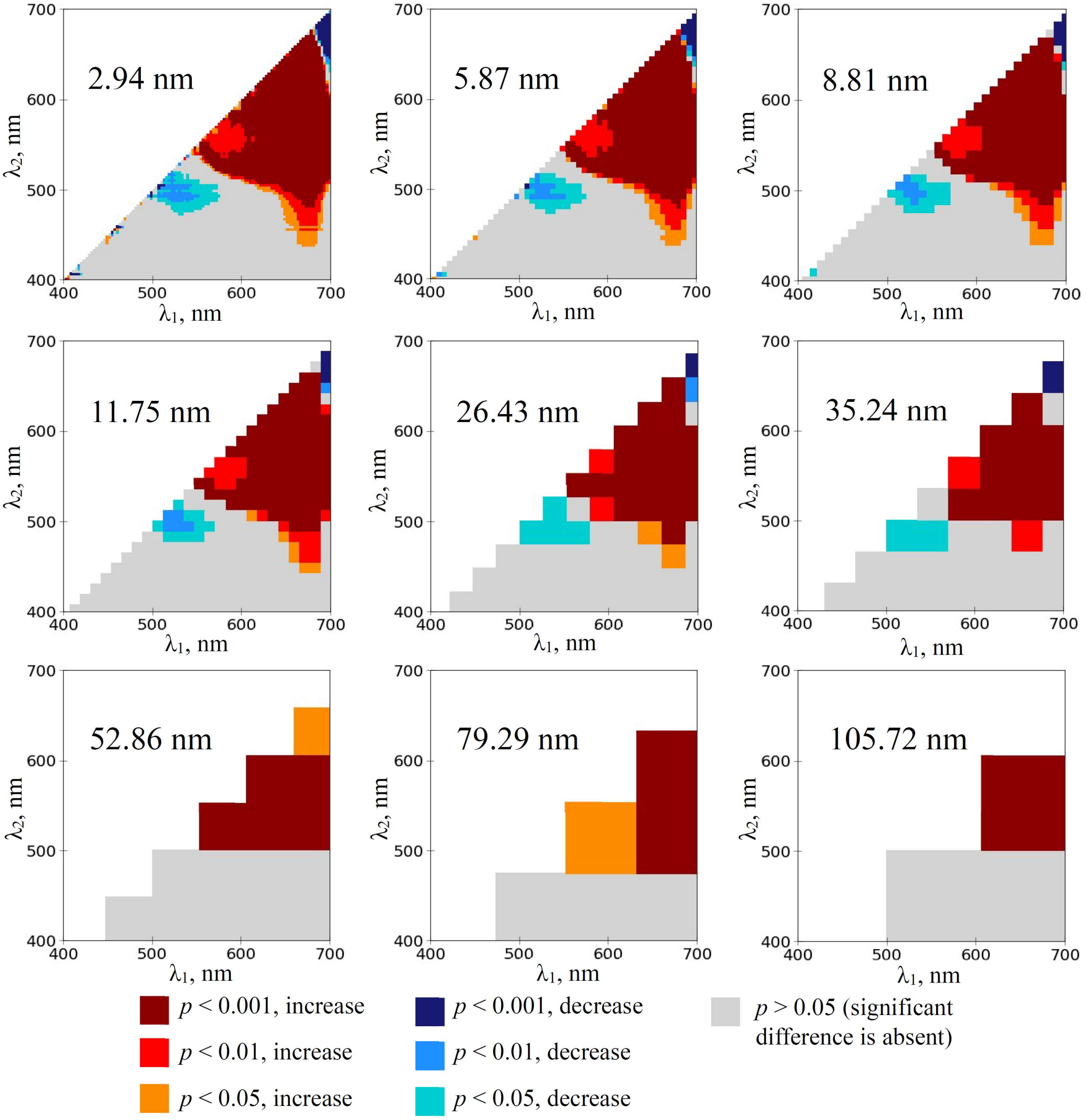

Figure S5. The heat maps of significance and direction of difference between normalized difference reflectance indices (RIs) in wheat plants under drought and control conditions on the 3rd day of drought (*n*=10). The spectral bandwidths are shown on maps. The significance and direction of changes in RIs are shown by colors.

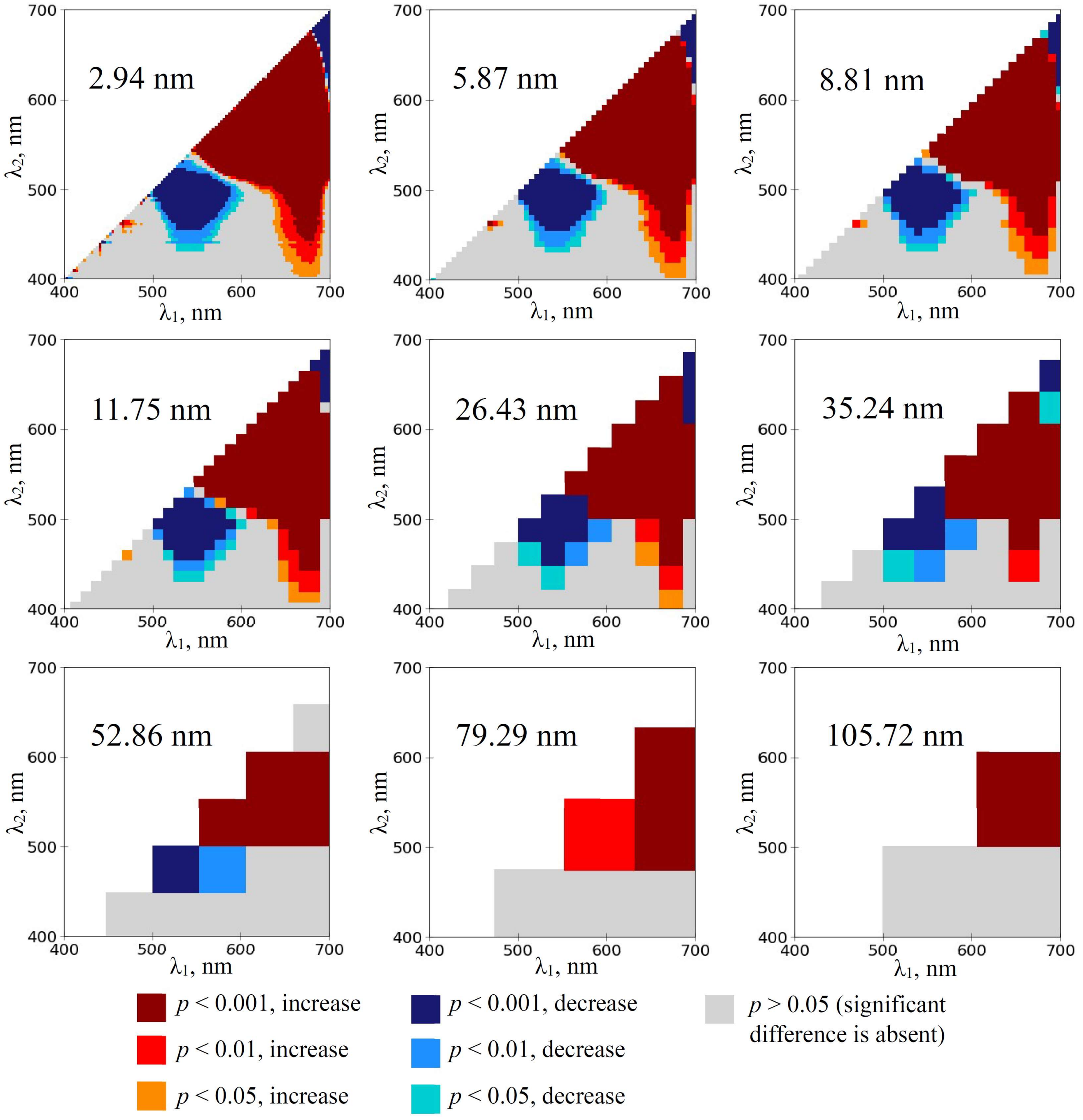

Figure S6. The heat maps of significance and direction of difference between normalized difference reflectance indices (RIs) in wheat plants under drought and control conditions on the 5th day of drought (n=10). The spectral bandwidths are shown on maps. The significance and direction of changes in RIs are shown by colors.

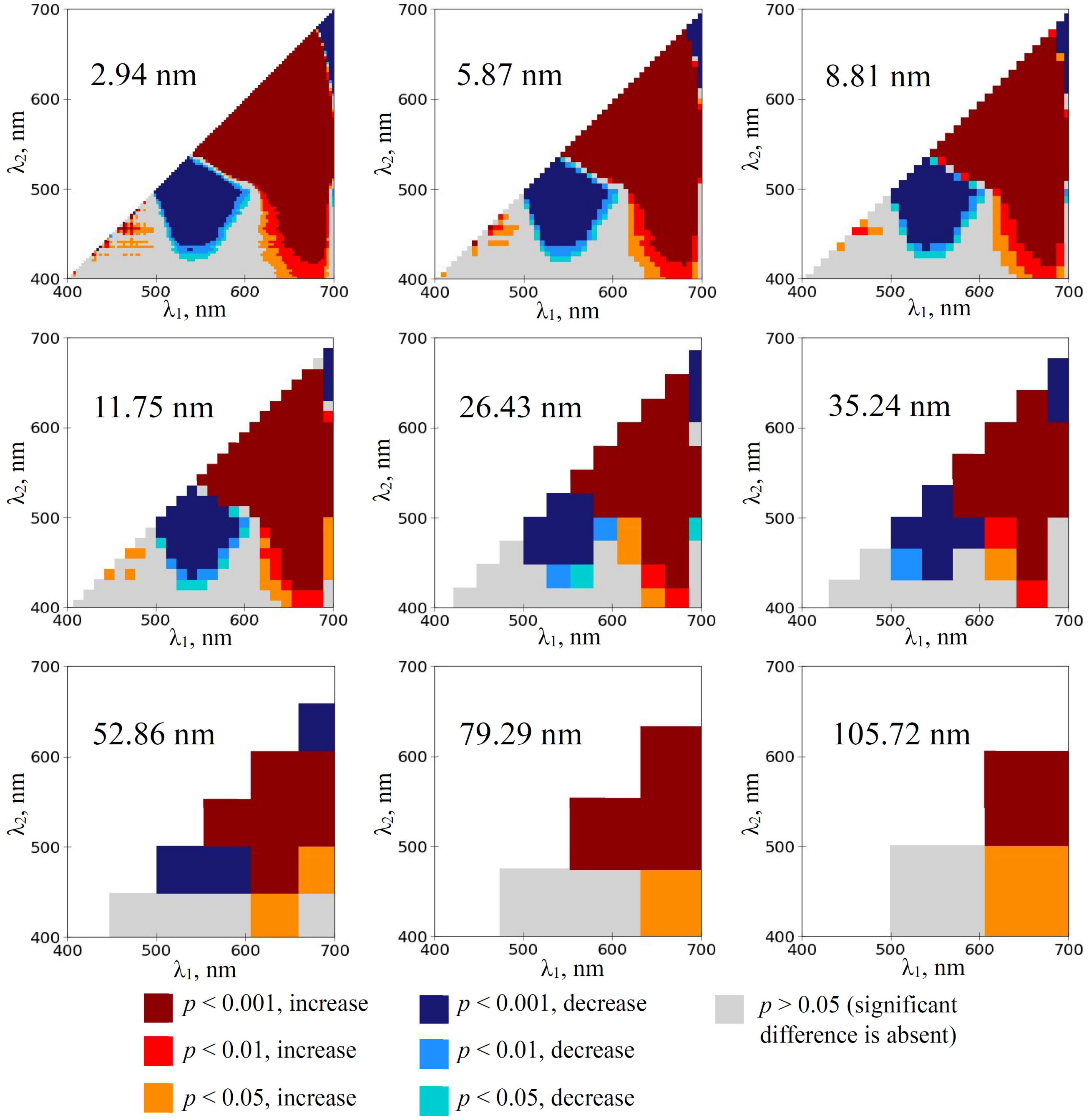

Figure S7. The heat maps of significance and direction of difference between normalized difference reflectance indices (RIs) in wheat plants under drought and control conditions on the 8th day of drought (n=10). The spectral bandwidths are shown on maps. The significance and direction of changes in RIs are shown by colors.

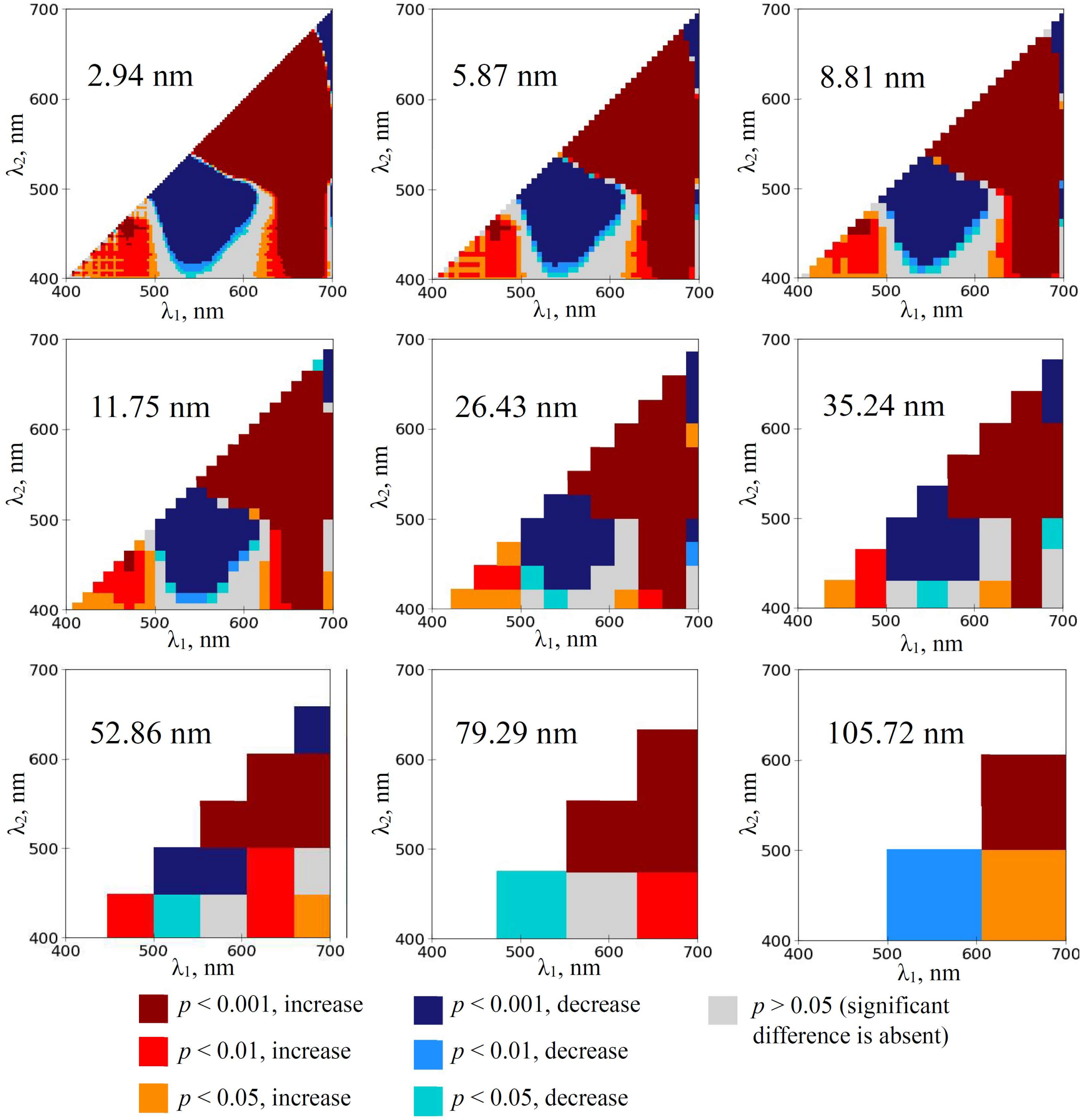

Figure S8. The heat maps of significance and direction of difference between normalized difference reflectance indices (RIs) in wheat plants under drought and control conditions on the 10th day of drought (*n*=10). The spectral bandwidths are shown on maps. The significance and direction of changes in RIs are shown by colors.

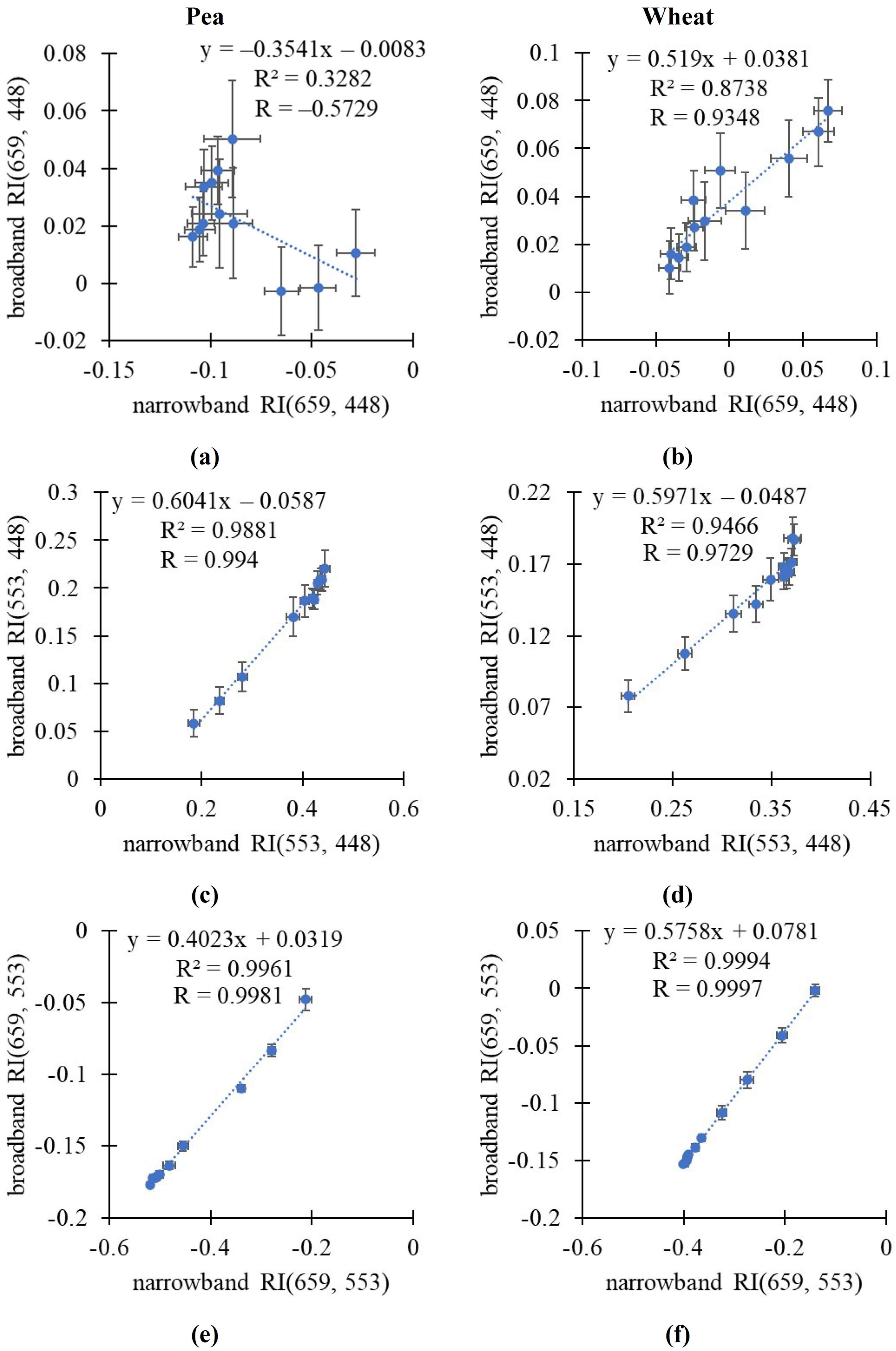

Figure S9. The scatter plots between broadband and narrowband normalized difference reflectance indices in pea and wheat plants under drought and control conditions. Scatter plots between broadband RI(659, 448) and narrowband RI(659, 448) in pea (**a**) and wheat (**b**), between broadband RI(553, 448) and narrowband RI(553, 448) in pea (**c**) and wheat (**d**), and between broadband RI(659, 553) and narrowband RI(659, 553) in pea (**e**) and wheat (**f**) are shown. Narrow bandwidth was 2.94 nm; broad bandwidth was 105.72 nm. Average values of indices were used. R^2 and R are determination and correlation coefficients.
